# A novel recombinant cell fluorescence biosensor based on toxicity of pathway for rapid and simple evaluation of DON and ZEN

**DOI:** 10.1038/srep31270

**Published:** 2016-08-08

**Authors:** Jian Ji, Wenshu Gu, Chao Sun, Jiadi Sun, Hui Jiang, Yinzhi Zhang, Xiulan Sun

**Affiliations:** 1 State Key Laboratory of Food Science and Technology, School of Food Science of Jiangnan University, School of Food Science Synergetic Innovation Center of Food Safety and Nutrition, Wuxi, Jiangsu, 214122, China

## Abstract

During an exposure, humans and animals are most often exposed to a mixture rather than individual mycotoxins. In this study, a Human Embryonic Kidney 293 cell (HEK-293) fluorescence sensor was developed to detect and evaluate mycotoxins, deoxynivalenol (DON) and zearalenone (ZEN) compounds, produced by *Fusarium culmorum* that are common food contaminants. TRE-copGFP (green fluorescent protein) and ERE-TagRFP (red fluorescent protein) plasmids were constructed and cotransfected into HEK-293 cells through a highly efficient, lipid-mediated, DNA-transfection procedure. Results show that fluorescence intensity was proportional to DON and ZEN concentrations, ranging from 2 to 40 ng/mL and 10 to 100 ng/mL respectively, with a detection limit of 0.75 ng/mL and 3.2 ng/mL respectively. The EC_50_ of DON and ZEN are 30.13 ng/mL and 76.63 ng/mL respectively. Additionally, ZEN may have a synergistic effect on enhancing AP-1 activity of the toxicity pathway of DON. These data indicate the high sensitivity and effectiveness of our biosensor system in the evaluation of the combined toxicity of ZEN, DON and their derivatives. In addition, this approach is suitable for an early warning method for the detection of ZEN and DON family mycotoxins contamination without higher-priced, conventional analytical chemistry methods.

Mycotoxins are compounds produced by mold fungi under moist conditions. Approximately 25% of the world’s crops are contaminated with mould or fungal growth and mycotoxins may be produced both before and after harvest^1^. In both humans and animals, the ingestion of food or feed contaminated by mycotoxins can lead to mycotoxicoses, the possible symptoms of which are acute intoxication, losses in productivity, reduced weight gain, immunosuppression and increased risk of cancer^2^.

Deoxynivalenol (DON), a representative mycotoxin of the trichothecene B group, is one of the most widespread cereal contaminants worldwide^3^. DON can be degraded or detoxified into various derivatives, such as 3-acetyl-DON and 15-acetyl-DON, by acetylation, oxidation, de-epoxidation, or glycosylation^4^,^5^,^6^,^7^. Numerous studies have addressed the toxicity of DON and its derivatives in animals^8^,; swine are the most susceptible species^9^,^10^. At the cellular level, the trichothecene DON and its derivatives disrupt normal cell function by binding to the ribosome and inhibiting protein synthesis and by activating cellular kinases involved in signal transduction^11^. DON-induced toxicity was previously suggested to involve the AP-1 family of transcription factors^12^. DON alone was able to induce AP-1 binding activity, and the induction involved a major activation of the c-Jun and c-Fos components^13^. Further, AP-1 binding was found to precede the expression of inflammatory cytokines, suggesting its importance in DON-induced immunostimulatory effects^14^,^15^. AP-1 was one of the first mammalian transcription factors to be identified, and regulates a wide range of cellular processes, including cell proliferation, death, survival and differentiation^16^. AP-1 regulates transcription of genes through its ability to bind specifically to the recognition site 5′-TGANTCA-3′, also known as the TPA (12-O-tetradecanoyl phorbol 13-acetate) response element (TRE)^17^.

The mycotoxin zearalenone is produced by *Fusarium* species as well as the metabolites zearalanone, α-zearalanol and β-zearalanol. α-zearalenol and β-zearalenol are exert harmful heath effect via their strong estrogenic activities, resulting in decreased fertility, increased fetal resorption, and changes in the weight of endocrine glands and serum hormone levels^18^. These compounds have a high relative binding affinity for estrogen receptor and exhibit high transactivation activity^19^, acting through Ers^20^,^21^,^22^ to activate the transcription of estrogen-responsive genes both *in vivo*^23^,^24^,^25^ and *in vitro*^26^,^27^. They are competitive inhibitors for the estrogen hormone leading to problems in the mammalian reproductive system^28^. Estrogen receptors belong to the steroid–thyroid hormone receptor superfamily and are located in the nucleus of the cell. In their inactive form they are associated with heat shock proteins in a multi-protein complex^29^. Binding of substances to the ligand-binding pocket of the estrogen receptor leads to its activation. The activated, ligand-bound estrogen receptor dissociates from the multi-protein complex, dimerizes, and moves to the nucleus, where it can bind to an estrogen response element (ERE), sequences in the promoter regions of estrogen target genes. Binding of the activated estrogen receptor dimer to these promoter elements regulates the transcription of these genes^30^.

Biological assays of cell activity and viability evaluation, such as by measuring the mitochondrial reduction of tetrazolium salts into an insoluble dye (the MTT test), the release of the enzyme lactate dehydrogenase (LDH), the measurement of reactive oxygen species (ROS), or mitochondrial apoptosis can be used to evaluate toxicity^31^. These methods require specific reagents, significant sample preparation time, and biological expertise. Moreover, as these are end-point assays, they cannot provide assessment of the cells’ recovery or long-term survival. Methods based instead on engineering cells to report toxicity by monitoring fluorescence has the potential to be reagent-free, simple, and nondestructive. Genetically modified (GM) yeast cells have been successfully used to evaluate toxicity^32^,^33^,^34^,^35^,^36^,^37^, estrogenicity^38^,^39^,^40^,^41^,^42^,^43^ and androgenicity of compounds^44^,^45^, including other endocrine-disrupting compounds mimicking hormone-like molecules.

Transcriptional regulation is a primary cellular strategy to control gene expression in response to either physiological or environmental stress signals^46^. These signals can activate a network of signal transduction pathways and subsequently induce relevant transcription factor activities. Activated transcription factors coordinately modulate target gene expression via binding to specific DNA sequences located within the promoter or enhancer. According to the transcriptional pathway of trichothecene DON and ZEN, we have developed a new cell-based biosensor system for the rapid and simple evaluation of mycotoxins. This assay is based on the stable cotransfection of HEK293 cells with two plasmids that separately encode the green fluorescent protein GFP reporter gene under the transcriptional control of the TRE promoter (TRE-GFP) and the red fluorescent protein RFP reporter gene under the transcriptional control of ERE promoter (ERE-RFP). We measured changes in fluorescence intensity induced by the mycotoxins in HEK293 cells and analyzed the relationship between mycotoxin concentrations and fluorescence signals. These HEK293 cell sensors successfully identified and evaluated target mycotoxins and the reaction process was monitored in real time by confocal laser scanning microscopy (CLSM), as shown in Fig. 1.

## Results

### Successful construction and expression of fluorescent protein

We amplified the TRE and ERE gene with oligonucleotides, so that Xba I and EcoR I restriction sites were introduced at the 5′ and 3′ ends, respectively. The PCR products of TRE and ERE and the vectors of pcopGFP and pTagRFP were also double-digested with Xba I and EcoR I, ligated with T4 DNA ligase respectively, and separately transfected into DH5a competent cells (Fig. 2A,C). The recombinant plasmids were double-digested with Xba I and EcoR I to check, and the positive clones were named pcopGFP-TRE and pTagRFP-ERE respectively (Fig. 2E). The anti-GFP and anti-RFP antibodies were used to detect GFP and RFP fluorescent protein expression.

### The measurement of ROS production and cell apoptosis

FACS analysis were performed to investigate potential adverse effects during transfection. Flow cytometric analyses were conducted for the cotransfection and control groups, using 6-carboxy-2,7′-diclorodihydrofluorescein diacetate, di(acetoxy ester) (DCFH-DA) (Molecular Probes, Eugene, OR) as a marker for ROS production and annexin V-FITC and PI as markers for apoptosis and necrosis, respectively (in the Figure S3).

### Optimization of the cell-based biosensor system

Due to a different mechanism of cell toxicity for DON and ZEN, to determine the earliest and stable expression time of different fluorescence proteins, we exposed the biosensor cells to culture medium containing DON (30 ng/mL) or ZEN (60 ng/mL). A sample without mycotoxin was used as a control. We measured the fluorescence intensity every 1 h after exposure. The fluorescence response of the biosensor cells was determined at different time points after exposure using high content screening. Image analysis was performed using the MetaXpress image analyzer (Molecular Devices, USA). We also used high content screening (HCS) to monitor cell responses after stimulation with DON (30 ng/mL) or ZEN (60 ng/mL) and measured fluorescence intensity changes over time to determine the amplitude and speed of the cellular responses, in Fig. 3.

### Quantification of DON and ZEN by HEK293 cell sensors

Figure 4 shows differential expression of GFP or RFP reporter proteins depending on mycotoxin-induced cell toxicity as a function of DON/ZEN dose. Compared to the control, GFP or RFP expression in a single cell increased as the DON/ZEN dose increased (Fig. 3A). Figure 4B clearly represents DON/ZEN dose-dependent cell toxicity.

### Quantification of combinations of DON and ZEN by HEK293 cell sensors

To estimate the cytotoxic interactions of DON and ZEN, the cell-based biosensors were treated with various concentrations and combinations of DON and ZEN as follows: DON + ZEN: 0.5 + 1, 1 + 2, 2+5, 5 + 10, 10 + 20, 20 + 40, 30 + 60, 40 + 80, 50 + 100 and 60 + 120 ng/mL. From Fig. 5A we found that every combination tested of these mycotoxins induced the expression of both fluorescent protein and the merged images revealed that DON/ZEN-induced toxicity occurred in both GFP and RFP reporter genes. Compared to the effect of mycotoxins taken separately, the fluorescence intensity of RFP did not change significantly, however, the fluorescence intensity of GFP increased, especially at high concentrations (Fig. 5B).

### Quantification of the derivatives of DON and ZEN by HEK293 cell sensors

To assay the response of the cell-based biosensor to the derivatives of DON and ZEN, we exposed it to a certain concentrations of derivatives of DON and ZEN. Figure 6A is a graph that represents the dose-dependent cell toxicity for the derivatives of DON and ZEN. EC_50_ values were calculated from the dose response curves. The EC_50_ of zearalenol (ZOL) and α-zearalanol (α-ZAL) was 20.93 ng/mL 42.63 ng/mL respectively (Fig. 6B).

## Discussion

**Figure 1:**
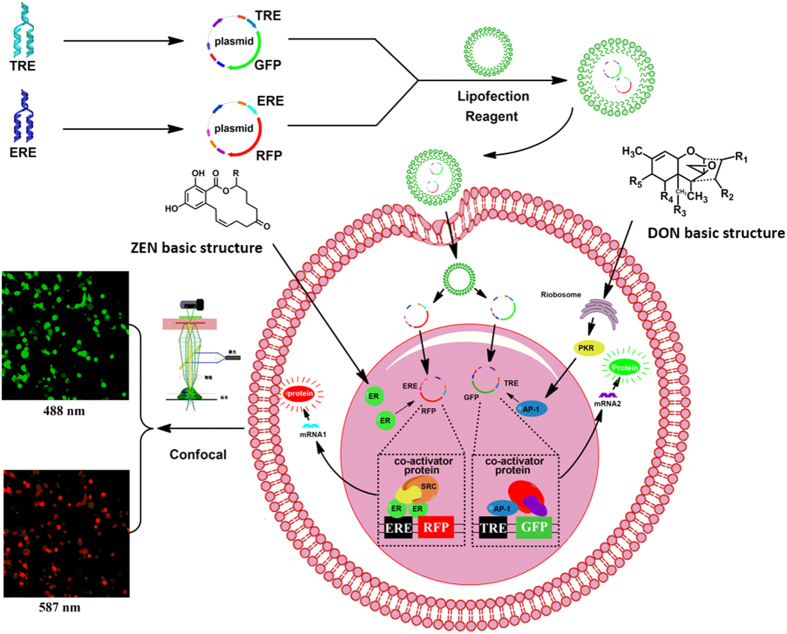
Schematic illustration of the working principle of a HEK293 cell sensor. TRE-GFP and ERE-RFP plasmids were introduced into HEK293 cells to obtain stable cotransfectant fluorescent HEK293 cells. When the biosensor cells were exposed to DON and ZEN, activation of transcription factors AP-1 and ER occurs, which then triggers the promoter of TRE and ERE respectively, finally leading to the expression of GFP and RFP fluorescent proteins. Therefore, mycotoxins are detected by the elevation of intracellular fluorescence emitted by GFP and RFP expression in HEK293 cells.

Figure 1 illustrates the mechanism by which a HEK293 cell sensor recognizes and quantifies DON and ZEN. We constructed two eukaryotic expression plasmids that encode the copGFP reporter gene under the transcriptional control of the TRE promoter (TRE-copGFP) and RFP reporter geen under the transcriptional control of ERE promoter (ERE-TagRFP) respectively. Then, we introduced the two fusion plasmids TRE-copGFP and ERE-TagRFP into HEK293 cells through a highly efficient, lipid-mediated, DNA-transfection procedure. DNA-lipid complexes were fused with the membrane by endocytosis, and the fluorescent protein gene was diffused throughout the intracellular membranes, thus entering the nucleus. Next, HEK293 cells were selected with the antibiotic G418 to obtain stable transfectant cells. When the biosensor cells were exposed to DON and ZEN, this stimulated activation of the transcription factors AP-1 and ER specifically, which stimulated the promotesr of TRE and ERE, finally leading to expression of the fluorescent proteins copGFP and TagRFP. In this way, we monitored cell responses and quantified the mycotoxin levels by detecting the intracellular fluorescence changes using a CLSM microscope.

**Figure 2:**
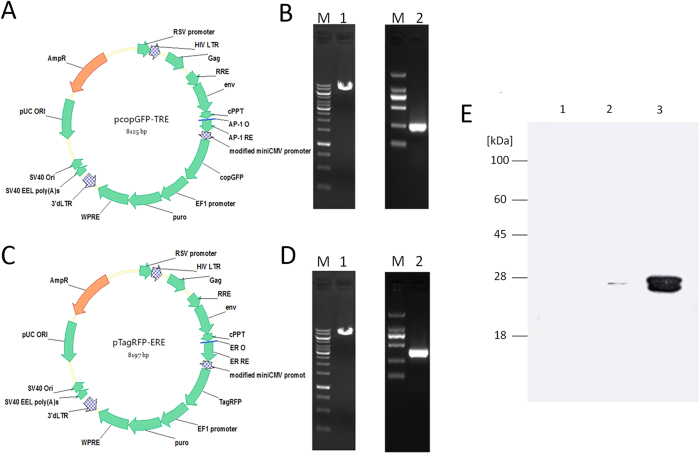
Construction of the pcopGFP-TRE and pTagRFP-ERE plasmids and expression of the GFP and RFP protein. (**A**) Map of the pcopGFP-TRE plasmid. The TRE gene (not including the stop codon) was introduced at the Xba I and EcoR I restriction sites of the multiple cloning site (MCS) region of the pcopGFP vector. (**B**) pcopGFP vector were digested with Xba I and EcoR I. where M represents DNA markers (10 kb, 8 kb, 6 kb, 5 kb, 4 kb, 3.5 kb, 3 kb, 2.5 kb, 2 kb, 1.5 kb, 1 kb, 750 bp, 500 bp, 250 bp) and 1 represents the pcopGFP-TRE plasmid doubly digested with Xba I and EcoR I (left). The TRE amplified by PCR with specific primers. Where M represents DNA markers (2 kb, 1 kb, 750 bp, 500 bp, 250 bp, 100 bp) and 1 represents the PCR product of TRE (right). (**C**) Map of the pTagRFP-ERE plasmid. ERE gene (not including the stop codon) was introduced at the Xba I and EcoR I restriction sites of the multiple cloning site (MCS) region of the pTagRFP vector; (**D**) pTagRFP vector were digested with Xba I and EcoR I. where M represents DNA markers (10 kb, 8 kb, 6 kb, 5 kb, 4 kb, 3.5 kb, 3 kb, 2.5 kb, 2 kb, 1.5 kb, 1 kb, 750 bp, 500 bp, 250 bp) and 1 represents the pTagRFP -ERE plasmid doubly digested with Xba I and EcoR I (left). The ERE was amplified by PCR with specific primers. Where M represents DNA markers (2 kb, 1 kb, 750 bp, 500 bp, 250 bp, 100 bp) and 1 represents the PCR product of ERE (right). (**E**) Western blotting of lysates prepared from HEK293 cells at 48 h after cotransfection with the pcopGFP-TRE and pTagRFP-ERE plasmids with anti-GFP and anti-RFP antibodies: 1, negative control (non-transfection cell); 2, control (cell contransfected with plasmids at 48 h); 3, positive control (cell cotransfected with plasmids treated with 30 ng/mL DON and 60 ng/mL ZEN).

Possibly because GFP and RFP fluorescent protein molecular sizes were too similar, two major bands, nearly 27 kDa (the GFP and RFP fluorescent proteins), were observed (Fig. 2E), good agreement with the molecular sizes of GFP (26.9) and RFP (27.6) respectively. The HEK293 cells infected with TRE-GFP showed strong fluorescence signals from GFP expression after three days due to its overexpression, indicating a high transfection efficiency of about 89.36 ± 2.37% as measured by fluorescent observation and flow analysis (see Figure S1 of the Supporting Information).

There were no significant differences in ROS production between the cotransfection and control groups (see Figure S2 of the Supporting Information). Similarly, the low rates of early apoptosis remained unchanged (1.42 ± 0.24 versus 2.16 ± 0.21%, respectively) and necrosis (0.79 ± 0.16 versus 1.47 ± 0.19%, respectively; see Figure S3 of the Supporting Information).

**Figure 3:**
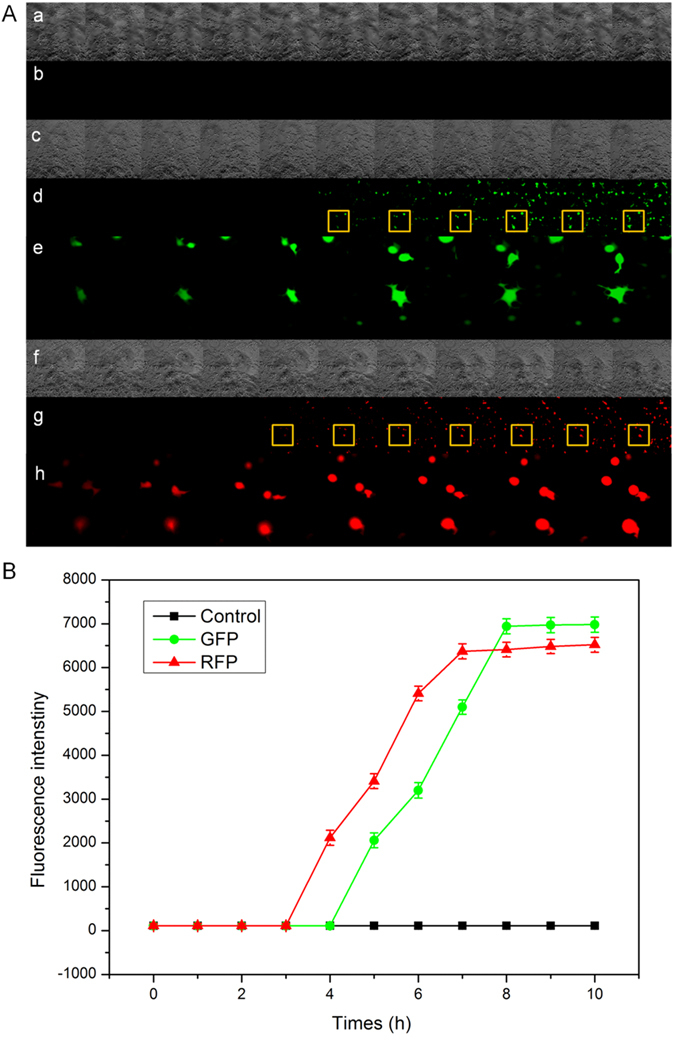
Fluorescence intensity changes of HEK293 cells exposed to certain concentrations of DON (30 ng/mL) and ZEN (60 ng/mL). (**A**) Time-dependent images of cell fluorescence intensity responses. DON (30 ng/mL) or ZEN (60 ng/mL) were used to stimulate HEK-293 biosensor cells. The images were recorded every 1 h. Control: (a) bright field (b) dark field, DON: (c) bright field, (d) GFP field, (e) Magnified image of the portion of (d) marked with the yellow box, ZEN: (f) bright field, (g) RFP field, (h) Magnified image of the portion of (g) marked with the yellow box. Quantification of fluorescence intensity in HEK-293 biosensor cells after sensitization with (**B**) DON (30 ng/mL) or (**C**) ZEN (60 ng/mL).

DON and ZEN standards were used to evaluate the feasibility of HEK293 cell sensors. HCS images of HEK293 cell sensors responding to DON (30 ng/mL) and ZEN (60 ng/mL) revealed that activating HEK293 cells led to a rapid and robust increase in cell fluorescence intensity (Fig. 3A). From Fig. 3B,C, we can see that there was no significance response at earlier hours, but 4 h later, the expression of GFP and RFP increased as time increased, and both signals became stable nearly 8 h after exposure.

**Figure 4:**
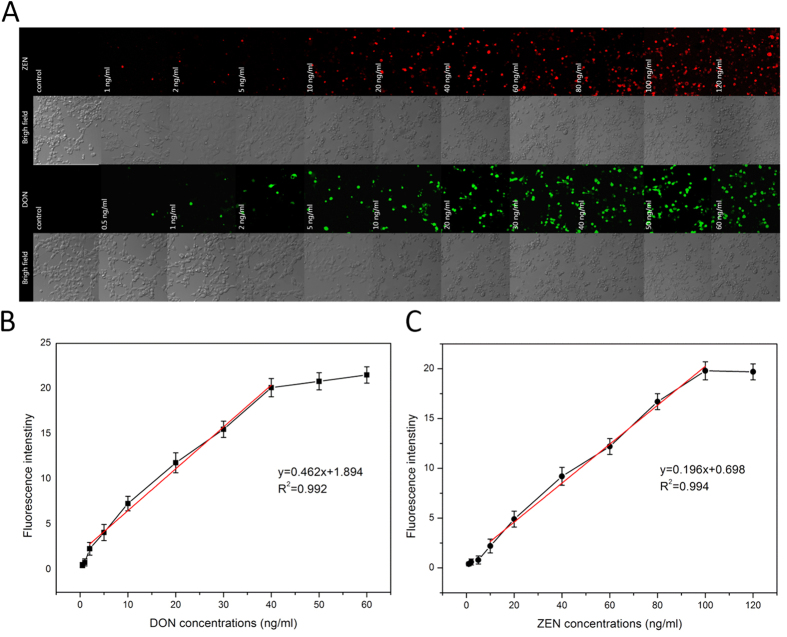
Quantification of DON and ZEN by HEK293-cell-based sensor. (**A**) Fluorescence intensity spectroscopy for HEK293 cells treated with different doses of mycotoxins. DON: 0.5, 1, 2, 5, 10, 20, 30, 40, 50, and 60 ng/mL, ZEN: 1, 2, 5, 10, 20, 40, 60, 80, 100 and 120 ng/mL. The curve of fluorescence intensity versus various concentrations of (**B**) DON and (**C**) ZEN. Data represent the mean ± SE of three different experiments under similar conditions.

DON was able to induce AP-1 binding activity, and the induction involved a major activation of the c-Jun and c-Fos components. AP-1 regulates transcription of genes through its ability to bind specifically to the TPA (12-O-tetradecanoyl phorbol 13-acetate) response element (TRE). This triggers the expression of fluorescent protein of GFP. ZEN has a very high relative binding affinity for the estrogen receptor. Once bound and activated, the ligand-bound estrogen receptor dissociates, dimerizes and moves to the nucleus, where it can bind to an estrogen response element (ERE). This results in the expression of fluorescent protein of RFP. As shown in the cellular images, the degree of mycotoxin-induced cell toxicity was visualized by fluorescent protein expression. EC_50_ values (the concentrations yielding half of the maximum response) were calculated from the dose response curves. The EC_50_ of DON and ZEN was 30.13 ng/mL and 76.63 ng/mL respectively. A linear relationship between the DON/ZEN concentrations and fluorescence intensity clearly shows that the fluorescence signals follow the same trend as that of mycotoxin-activated cells. Fluorescence intensity was proportional to DON and ZEN, ranging from 2 to 40 ng/mL and 10 to 100 ng/mL respectively. At both low and high DON/ZEN concentrations, fluorescence intensities exhibited good linear relationships with DON/ZEN concentrations, with correlation coefficients of 0.992 and 0.994 respectively. The limit of detection (LOD) calculated from Fig. 4B,C were 0.75 ng/mL and 3.2 ng/mL respectively, according to the formula: LOD = 3 s/m, where s represents the blank sample standard deviation (n = 3) and m represents the slope of the related DON/ZEN calibration curve. These measurements were performed in triplicate to assess the reproducibility and precision of freshly fabricated cell sensors. The relative standard deviation (RSD) of the detection results were all <5%, showing acceptable performance.

**Figure 5:**
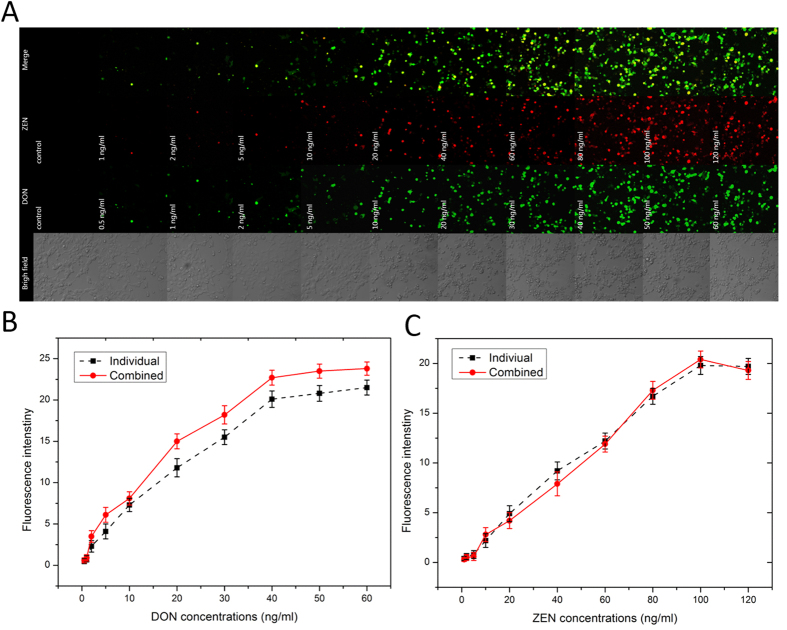
Quantification of the combination of DON and ZEN by HEK293-cell-based sensor. (**A**) Fluorescence intensity spectroscopy for HEK293 cells treated with various concentrations of combinations of DON and ZEN. DON + ZEN: 0.5 + 1, 1 + 2, 2 + 5, 5 + 10, 10 + 20, 20 + 40, 30 + 60, 40 + 80, 50 + 100 and 60 + 120 ng/mL. (**B**) The curve of fluorescence intensity versus various concentrations of combinations of DON and ZEN. Data represent the mean ± SE of three different experiments under similar conditions.

Most studies have evaluated the effect of mycotoxins taken individually. Nevertheless, it is very likely that humans and animals cold be exposed to a mixture of mycotoxins, rather than to individual compounds. This is particularly true for DON and ZEN, which are both be produced by *Fusarium culmorum* and are common contaminants that can co-occur in several cereal grains. The western blot analysis confirmed that DON induced expression of GFP protein, ZEN induced expression of RFP protein, and their combination further increased the expression of GFP (Figure S4). This is likely because DON can enhance AP-1 activity by its toxicity pathway and ZEN has a very high binding affinity for estrogen receptor which can enhance AP-1 activity by two distinct mechanisms. Likely, anti-estrogen-liganded ER enhances AP-1 activity via interactions with corepressors^47^,^48^, leading to an intensive expression of fluorescent protein of GFP. That means ZEN have a synergistic effect on enhancing AP-1 activity of the toxicity pathway of DON. From the evaluation of fluorescence intensity of individual toxicity and combined toxicity, in Fig. 5, the synergistic effect on enhancing AP-1 activity of the toxicity pathway of DON by ZEN was noticeable. Nonetheless, DON evinced no significant intervention on ER signal pathway, as shown in Fig. 5B. Meanwhile, the western blot assay was performed to validate the result of fluorescence analysis (Figure S4).

**Figure 6:**
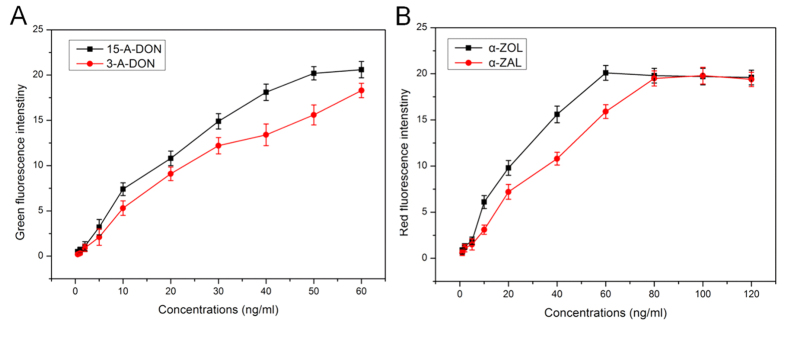
Quantification of the derivatives of DON and ZEN by HEK293 cell sensors. (**A**) The curve of fluorescence intensity versus various concentrations of combinations of 3-A-DON and 15-A-DON: 0.5, 1, 2, 5, 10, 20, 30, 40, 50, and 60 ng/mL. (**B**) The curve of fluorescence intensity versus various concentrations of combinations of α-ZOL and α-ZAL: 1, 2, 5, 10, 20, 40, 60, 80, 100 and 120 ng/mL. Data represent the mean ± SE of three different experiments under similar condition.

From Fig. 6, we can see the derivatives of DON can induce green fluorescence. EC_50_ values were calculated from the dose response curves. The EC_50_ of 15-A-DON and 3-A-DON was 31.65 ng/mL and 40.34 ng/mL, respectively. In this study, we observed that 3-ADON was less toxic to the HEK293 cells biosensor than 15-A-DON and DON. This result confirms the lower toxicity of 3-A-DON observed in other previous studies^49^,^50^,^51^ even though the difference in toxicity between 3-A-DON and the two other trichothecene varies in different reports. When the cell-based biosensor was exposed to α-ZOL, there was a significant induction in red fluorescence, suggesting that α-ZOL has very high estrogenic activity. Compared with ZEN, the estrogenic potencies of α-ZOL and α-ZAL were more powerful. This result differs from these^52^ reported that the compounds showed similar potency. The difference in these findings could be due to variations in the assay methods or the experimental conditions.

## Conclusion

We have develop a cell-based biosensor system that can report toxic stress caused by two kinds of mycotoxins: zearalenone family mycotoxins and deoxynivalenol family mycotoxins. The EC_50_ of DON and ZEN is 30.13 ng/mL and 76.63 ng/mL with a detection limit of 0.75 ng/mL and 3.2 ng/mL respectively. We found that ZEN may have a synergistic effect on enhancement of AP-1 activity of the toxicity pathway of DON. The biosensor cells can be assayed in a high-throughput, noninvasive manner, with no need for sophisticated equipment or reagents. This open-source biosensor can serve as an important resource for users who wish to evaluate the effect of mycotoxins on cell toxicity. This toxicity assay can be applied to the fields of mycotoxin evaluation and environmental and occupational monitoring of exposure to zearalenone and deoxynivalenol compounds and their complex mixtures.

## Methods

### Reagents and Chemicals

HEK-293 cells were obtained from the Cell Bank of Chinese Academy of Sciences (Shanghai, China). Dulbecco’s Modified Eagle’s Medium (DMEM) and fetal bovine serum (FBS) were obtained from Gibco Laboratories (Gaithersburg, MD). Glass-bottomed dishes (35 mm) were purchased from Shengyou Biotechnology Co., Inc. (Hangzhou, China). Other reagents were purchased from Sinopharm Chemical Reagent Co., Ltd. (Shanghai, China). All solutions were prepared with deionized water, and all reagents were of analytical grade.

### Instrumentation

Fluorescence signals were recorded by confocal laser scanning microscopy (CLSM, LSM 710, Carl Zeiss Microscopy GmbH, Göttingen, Germany). A FACS Calibur flow cytometer (BD Biosciences, San Jose, CA) was used to measure intracelluar reactive oxygen species and cell apoptosis. HEK-293 cells were incubated in a CO_2_ incubator (Thermo Scientific Forma Series II Water Jacket, Thermo Fisher Scientific, Inc., Waltham, MA). The fluorescence response of the biosensor cells was determined at different time points after exposure by high content screening (ImageXpress Micro XLS, Molecular Devices, USA).

### Construction of a TRE-copGFP and ERE-TagRFP prokaryotic expression system

The eukaryotic expression plasmids that encodes the copGFP reporter gene under the transcriptional control of the TRE promoter (TRE-copGFP) and the TagRFP reporter gene under the transcriptional control of the ERE promoter were prepared using standard molecular biology techniques of restriction and ligation. The sequence of the TRE and ERE promoters were cloned as described previously^53^,^54^ and amplified by PCR with specific primers: TRE: 5′-acc gta cac gcc taa agc gaa tt-3′ (forward) and 5′-cac cat tat cgt ttc aga ccc ac-3′ (reverse). ERE: 5′-ttt atc gat ttc tag att tac gtc ag-3′ (forward) and 5′-acc gta cac gcc taa agc tt-3′ (reverse). The TRE PCR products and pcopGFP vector were digested with Xba I and EcoR I, and the ERE PCR products and pTagRFP vector were digested with Xba I and EcoR I. The resulting fragments were purified and ligated with T4 DNA ligase. The ligated products were separately transformed into DH5a competent cells and the isolated positive clones were named pcopGFP-TRE and pTagRFP-ERE.

### Cotransfection of the recombinant plasmids

At 1 day before transfection, plates were prepared containing 2 × 10^5^ HEK-293 cells in 500 μL of growth medium without antibiotics, such that the cells were 90–95% confluent at the time of transfection. The ratio of cationic carrier/DNA was optimized on the basis of the instructions of the manufacturer. Briefly, 2 μg TRE-GFP and 2 μg ERE-RFP of recombinant plasmids were diluted in 50 μL of OptiMEM I reduced serum medium without serum (Gibco Invitrogen, Life Technologies) and mixed gently. A total of 10 μL of Lipofectamine2000 (Invitrogen, Life Technologies, Grand Island, NY) was mixed gently before use and added to 50 μL of Opti-MEM I medium. After 5 min incubation at room temperature, diluted recombinant plasmids were combined with the diluted Lipofectamine 2000 (total volume of 100 μL) and incubated for 20 min at room temperature. Then, the mixture was added to HEK-293 cell plates. After 4 h for the DNA transfection, the transfection medium was replaced with fresh culture medium and these cells with the TRE-copGFP and ERE-TagRFP plasmids were cultured in 35 mm dishes for a few days. Then, stable transfectants with GFP and RFP were selected with the antibiotic puromycin (Gibco Invitrogen).

### Western Blot analysis for fluorescent protein expression

To each tube of harvested cells (in 20 μL), 180 μL of radio-immunoprecipitation assay (RIPA) lysis buffer containing phenylmethylsulfonyl fluoride (PMSF) was added and incubated for 10 min at room temperature (RT). Equal amounts of proteins were separated using 10% sodium dodecyl sulfate-polyacrylamide gel electrophoresis (SDS-PAGE) after boiling for 5 min in 4× loading buffer and transferred onto Immobilon-P membranes (Millipore Corp., Billerica, MA). The membranes were then incubated with anti-GFP antibody, anti-RFP antibody and a secondary antibody immunoglobulin G (IgG horseradish peroxidase (HRP) (Santa Cruz Biotechnology, Santa Cruz, CA). Immunoreactivity was detected using the enhanced chemiluminescence (ECL) detection system (GE Healthcare Bio-Sciences Corp., Piscataway, NJ). The target protein bands were quantified by scanning densitometry using ImageJ processing software and normalized to the signal intensity of GAPDH.

### Intracelluar reactive oxygen species (ROS) levels and apoptosis assay

Fluorescent probe 6-carboxy-2,T-diclorodihydrofluorescein diacetate, di(acetoxy ester) (DCFH-DA) (Molecular Probes, Eugene, OR) is non-fluorescent itself, and can cross the cell membrane freely. Intracellular DCFH-DA was hydrolyzed by esterase to produce DCFH, and intracellular ROS is able to oxidize non-fluorescent DCFH to generate fluorescent DCF. The transfected and untransfected HEK293 cells were loaded with 10 mM DCFH-DA and incubated for 20 min at 37 °C. The cells were then washed three times with serum-free medium to remove the extracellular DCFH-DA. Intracellular ROS levels in the transfected and untransfected cells were detected by determining the fluorescence intensity of DCF using a FACS Caliburflow cytometer (BD Biosciences, San Jose, CA).

The effect of plasmid transfer on cell survival was examined by evaluating apoptosis via fluorescence-activated cell-sorting analysis, including an apoptosis detection kit (Nanjing KeyGen Biotech Co., Ltd., Shanghai, China). Briefly, HEK-293 cells in 12-well plates were harvested by trypsinization at the 3^rd^ day after infection with TRE-GFP and ERE-RFP plasmids. After neutralization by washing with culture media containing FBS and phosphate-buffered saline, the cells were resuspended in 100 μL of annexin V-FITC binding buffer and stained in the dark with 4 μL of annexin V-FITC (an apoptosis marker) and 4 μL of propidium iodide (PI, a necrosis marker) for 15 min at room temperature as a control. Next, 400 μL of annexin V-FITC binding buffer was added, and the samples were analyzed by flow cytometry and CLSM.

### Quantitative characterization of the sensor in response to mycotoxins exposure

HEK-293 cells were cultured in a flask in DMEM medium supplemented with 10% fetal calf serum, penicillin (100 μg mL^−1^), and streptomycin (100 μg mL^−1^) at 37 °C in a humidified atmosphere containing 5% CO_2_. After three 3 days, cells reached logarithmic growth phase and they were plated onto 35 mm glass-bottomed dishes (2 × 10^5^ cells/dish) to form a monolayer overnight. Cells were washed with PBS, and then the biosensor cells were exposed to culture medium mixed with various concentrations of DON (0.5–60 ng/mL), ZEN (1–120 ng/mL) and their combinations, for 8 h respectively, at which time the cells were washed and incubated in normal cell culture medium until further analysis. The cell responses were monitored using a LSM 710 CLS microscope (Carl Zeiss Microscopy GmbH). The control cells were placed outside the incubator for the same amount of time as the mycotoxin-exposed biosensor cells and were washed the same number of times with PBS. The total fluorescence intensity for each image was calculated using Scion Image software (Scion Corp., Frederick MD). Cells not exposed to mycotoxins served as controls.

## Additional Information

**How to cite this article**: Ji, J. *et al*. A novel recombinant cell fluorescence biosensor based on toxicity of pathway for rapid and simple evaluation of DON and ZEN. *Sci. Rep*. **6**, 31270; doi: 10.1038/srep31270 (2016).

## Supplementary Material

Supplementary Information
