# Supplementary material for: A novel recombinant cell fluorescence biosensor based on toxicity of pathway for rapid and simple evaluation of DON and ZEN
Source: Sci Rep. 2016 Aug 8;6:31270. doi: 10.1038/srep31270 (PMC4976381; doi:10.1038/srep31270)
Supplement: Supplementary Information [file srep31270-s1.doc]

**【Supplementary Material 】：**

**A novel recombinant cell fluorescence biosensor based on toxicity of pathway for rapid and simple evaluation of DON and ZEN**

**Jian Ji^1^, Wenshu Gu^1^, Chao Sun^1^, Hui Jiang^1^, Yinzhi Zhang^1^, Xiulan Sun^1^,***

**^1^****State Key Laboratory of Food Science and Technology, School of Food Science of Jiangnan University, School of Food Science Synergetic Innovation Center of Food Safety and Nutrition. Wuxi, Jiangsu, 214122, China**

**First author: Jian Ji (E-mail: jijianjndx@126.com)**

***Corresponding author: Xiulan Sun (E-mail:** [**sxlzzz@jiangnan.edu.cn**](mailto:sxlzzz@jiangnan.edu.cn)**)**

Figure S1. Efficiency of TRE-GFP transfection and influence on cell proliferation and survival. (A) a: morphology and density of HEK-293 cells after transfection; b: Fluorescent detection of expression rates of GFP at the same region; c: 0.12% in non-transfection group by flow analysis; d: 89.36% in the optimal transfection group by flow analysis. (B) The curve of cell proliferation and survival show no significant difference in cell number at 1 to3 days between transfected and control HEK-293 cells.

Figure S2. (A) FACS analysis of intracellular ROS in control (left) and transfection HEK-293 cells (right). The assay show no difference in intracellular ROS between transfected and control HEK293 cells.

Figure S3. (A) FACS analysis of apoptosis in control (left) and transfection HEK-293 cells (right). The lower left quadrant contains annexin V-FITC (-) and PI (-) viable cells; the lower right quadrant, annexin V-FITC (+) and PI (-) early apoptotic cells; the upper right quadrant, annexin V-FITC (+) and PI (+) late apoptotic or necrotic cells; the upper left quadrant, annexin V-FITC (-) and PI (+) necrotic cells. Bars indicate % of apoptotic or necrotic cells in each group expressed as mean ± SD of data obtained from three independent experiments. (B) The images of apoptosis in control (b) and transfection HEK-293 cells (c) recorded by CLSM. (a): Positive control: HEK-293 treated with 10 ug/ml DON.

Figure S4 After exposure of HEK293 cells to DON (30 ng/mL) ZEN (60 ng/mL) and their combinations for 8 h respectively, fluorescent protein GFP and RFP levels were examined respectively. The western blot analysis showed that DON and ZEN can induce the expression of GFP and RFP protein respectively. While their combinations can enhance the expression of GFP protein.

**The positive clone sequencing and characterization of TRE and ERE**

**
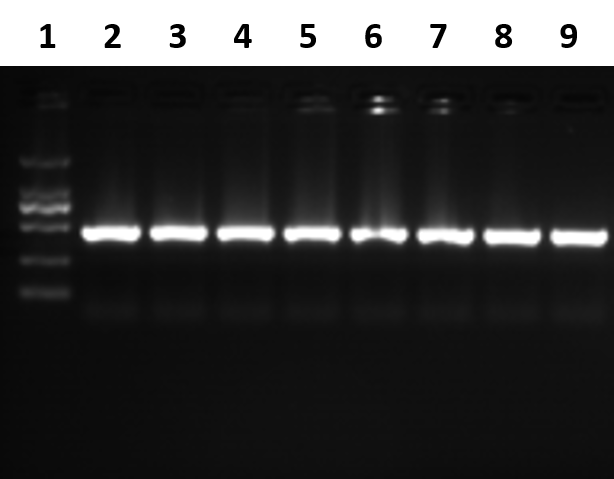
**

1:DL2,000 DNA Marker： 2 kb， 1 kb，750 bp，500 bp，250 bp，100 bp,

2-9: TRE-copGFP positive colony PCR identification map

The positive clone sequencing of TRE:


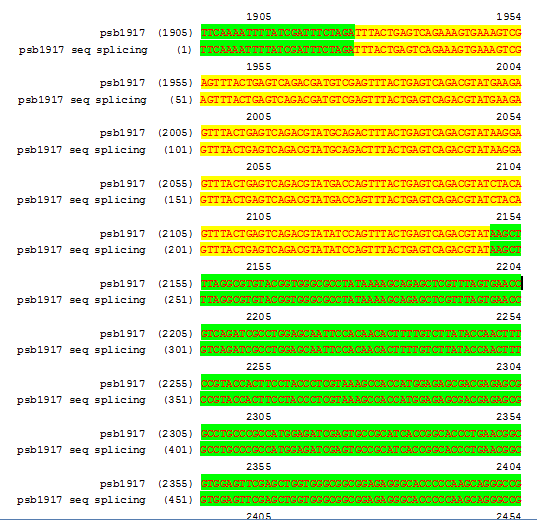


**The positive clone sequencing of ERE**

**
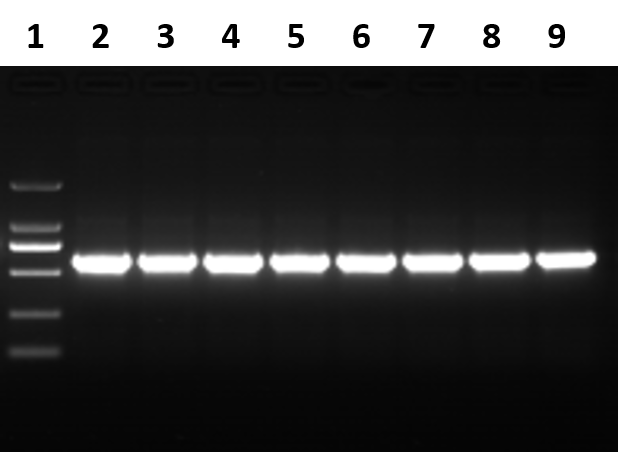
**

1：DL2,000 DNA Marker： 2 kb， 1 kb，750 bp，500 bp，250 bp，100 bp,

2-9： ERE-TagRFP positive colony PCR identification map

The positive clone sequencing of ERE:


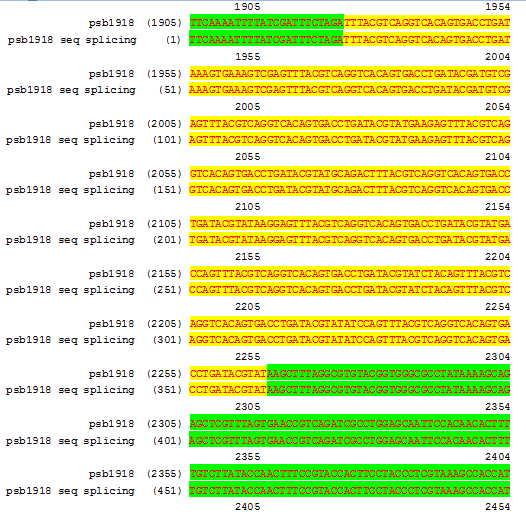


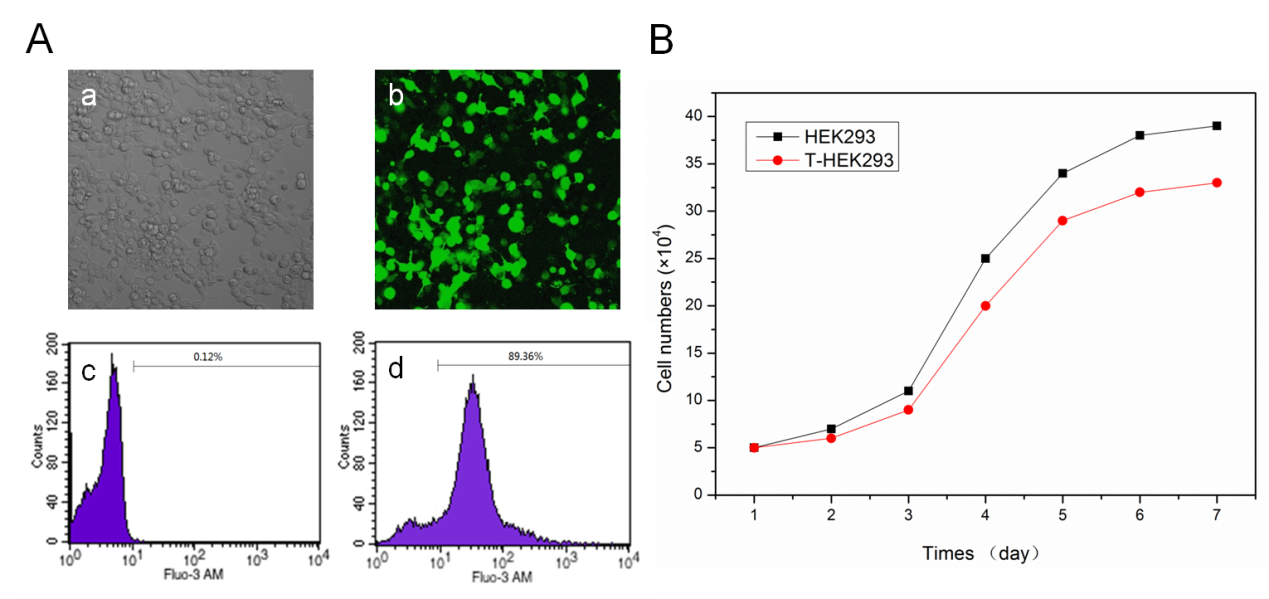


Figure S1. Efficiency of TRE-GFP transfection and influence on cell proliferation and survival. (A) a: Morphology and density of HEK-293 cells after transfection; b: Fluorescent detection of expression rates of GFP at the same region; c:0.12% in non-transfection group by flow analysis; d:89.36% in the optimal transfection group by flow analysis.(B) The curve of cell proliferation and survival show no significant difference in cell number at 1 to3 days between transfected and control HEK-293 cells.


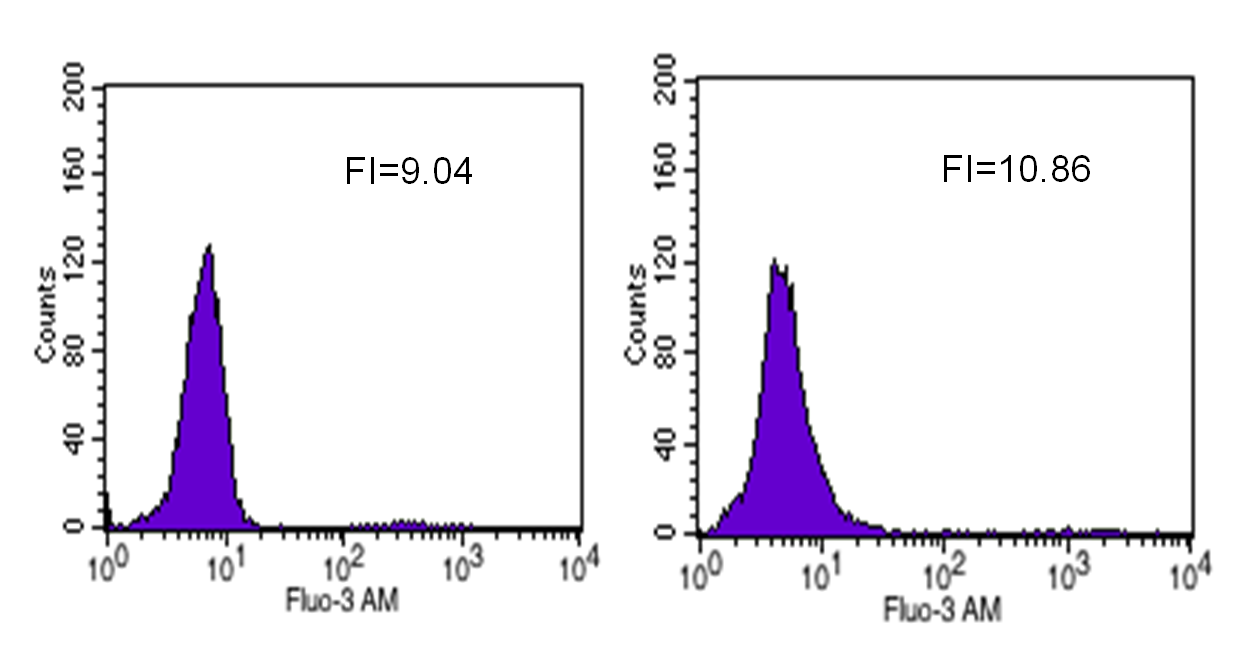


Figure S2. (A) FACS analysis of intracelluar ROS in control (left) and transfection HEK-293 cells (right). The assay show no difference in intracelluar ROS between transfected and control HEK293 cells.


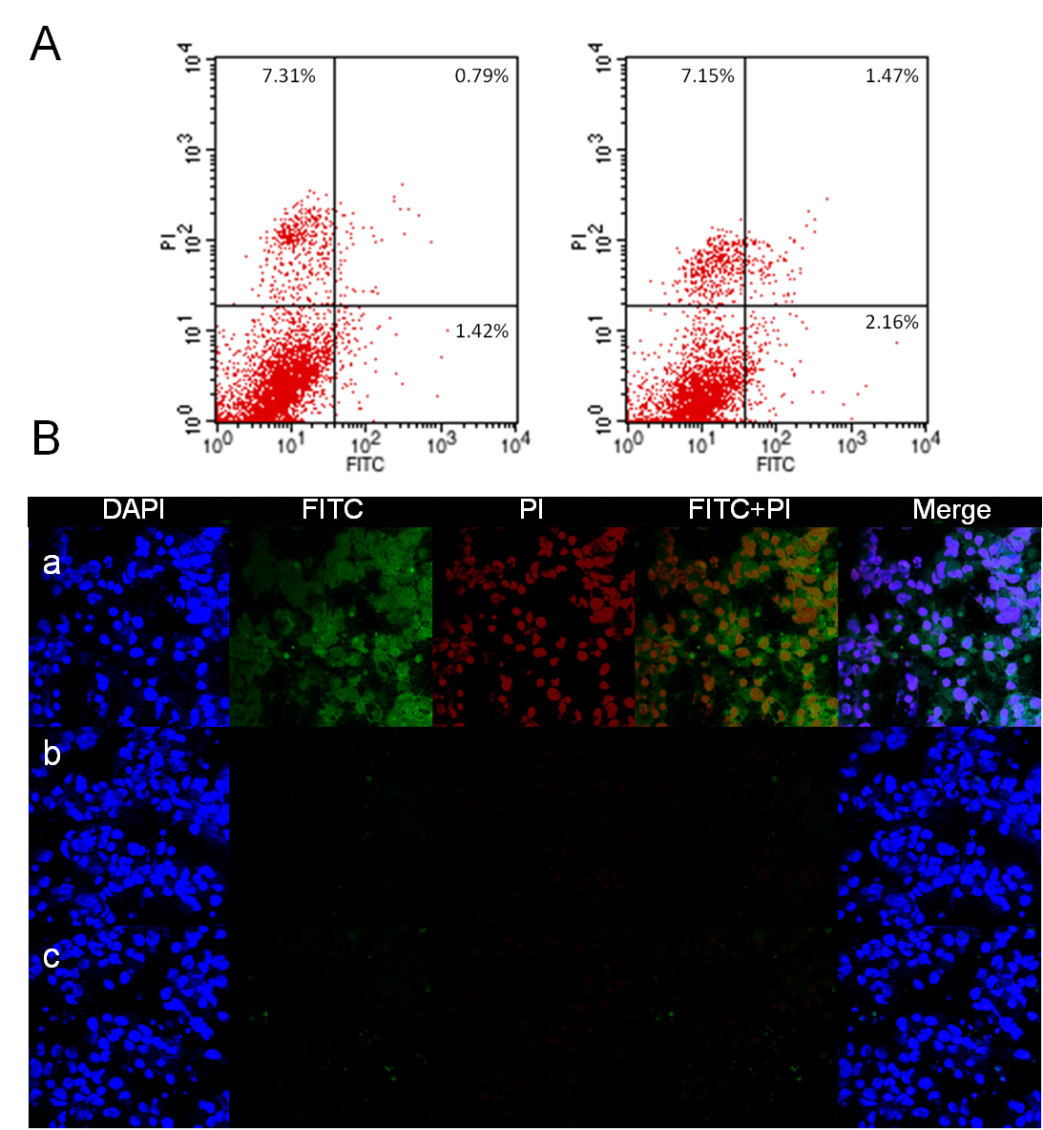


Figure S3. (A) FACS analysis of apoptosis in control (left) and transfection HEK-293 cells (right). The lower left quadrant contains annexin V-FITC (-) and PI (-) viable cells; the lower right quadrant, annexin V-FITC (+) and PI (-) early apoptotic cells; the upper right quadrant, annexin V-FITC (+) and PI (+) late apoptotic or necrotic cells; the upper left quadrant, annexin V-FITC (-) and PI (+) necrotic cells. Bars indicate % of apoptotic or necrotic cells in each group expressed as mean ± SD of data obtained from three independent experiments. (B) The images of apoptosis in control(b) and transfection HEK-293 cells(c) recorded by CLSM. (a): Positive control: HEK-293 treated with 10 ug/ml DON.


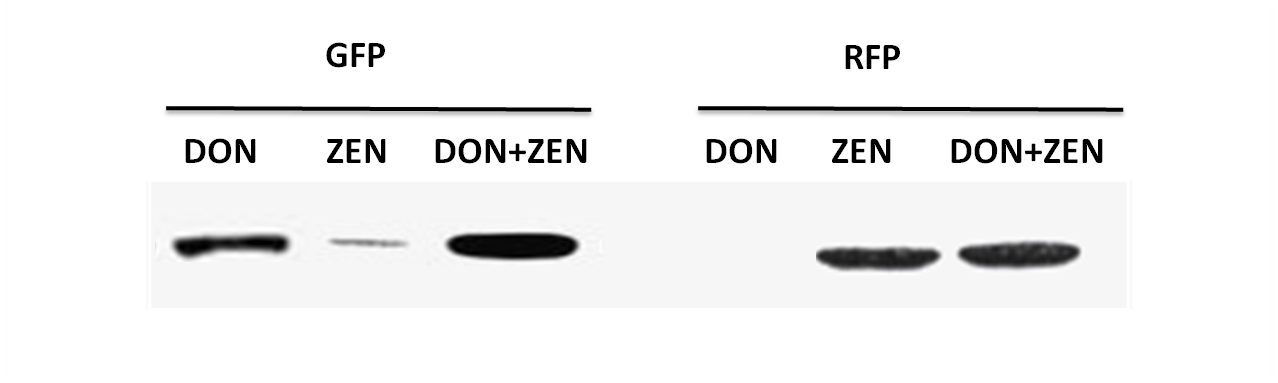


Figure S4 After exposure of HEK293 cells to DON (30 ng/mL) ZEN (60 ng/mL) and their combinations for 8 h respectively, fluorescent protein GFP and RFP levels were examined respectively. The western blot analysis showed that DON and ZEN can induce the expression of GFP and RFP protein respectively. While their combinations can enchance the expression of GFP protein.

**Reference**
